# Supplementary material for: Empirical methods for controlling false positives and estimating confidence in ChIP-Seq peaks
Source: BMC Bioinformatics. 2008 Dec 5;9:523. doi: 10.1186/1471-2105-9-523 (PMC2628906; doi:10.1186/1471-2105-9-523)
Supplement: Additional File 1 — A variety of html documents from the USeq web site detailing the available applications, their best usage, output file type descriptions, command line menus, etc. [file 1471-2105-9-523-S1.zip › USeqUserGuides/JavaDoc.zip!/JavaDoc/serialized-form.html]

Serialized Form


|  |  |  |
| --- | --- | --- |
| **Overview** \| Package \| Class \| Use \| **Tree** \| **Deprecated** \| **Index** \| **Help** | |  |
| PREV   NEXT | **FRAMES**    **NO FRAMES**     **All Classes** |  |


---


# Serialized Form


---

**Package** **bioroot**

**Class bioroot.AccountManagement extends javax.servlet.http.HttpServlet implements Serializable**

**Class bioroot.Archive extends javax.servlet.http.HttpServlet implements Serializable**

**Class bioroot.CellType extends javax.servlet.http.HttpServlet implements Serializable**

**Class bioroot.Contact extends javax.servlet.http.HttpServlet implements Serializable**

**Class bioroot.Gene extends javax.servlet.http.HttpServlet implements Serializable**

**Class bioroot.Login extends javax.servlet.http.HttpServlet implements Serializable**

**Class bioroot.Logout extends javax.servlet.http.HttpServlet implements Serializable**

**Class bioroot.Marker extends javax.servlet.http.HttpServlet implements Serializable**

**Class bioroot.Organism extends javax.servlet.http.HttpServlet implements Serializable**

**Class bioroot.Search extends javax.servlet.http.HttpServlet implements Serializable**

**Class bioroot.UserRegistration extends javax.servlet.http.HttpServlet implements Serializable**

---

**Package** **bioroot.antibody**

**Class bioroot.antibody.AntibodyBase extends javax.servlet.http.HttpServlet implements Serializable**

**Class bioroot.antibody.AntibodyModify extends javax.servlet.http.HttpServlet implements Serializable**

**Class bioroot.antibody.AntibodySpreadSheet extends javax.servlet.http.HttpServlet implements Serializable**

**Class bioroot.antibody.AntibodyViewPreferences extends javax.servlet.http.HttpServlet implements Serializable**

**Class bioroot.antibody.NewAntibody extends javax.servlet.http.HttpServlet implements Serializable**

---

**Package** **bioroot.oligo**

**Class bioroot.oligo.NewOligo extends javax.servlet.http.HttpServlet implements Serializable**

**Class bioroot.oligo.OligoBase extends javax.servlet.http.HttpServlet implements Serializable**

**Class bioroot.oligo.OligoModify extends javax.servlet.http.HttpServlet implements Serializable**

**Class bioroot.oligo.OligoSpreadSheet extends javax.servlet.http.HttpServlet implements Serializable**

**Class bioroot.oligo.OligoUpload extends javax.servlet.http.HttpServlet implements Serializable**

**Class bioroot.oligo.OligoViewPreferences extends javax.servlet.http.HttpServlet implements Serializable**

---

**Package** **bioroot.plasmid**

**Class bioroot.plasmid.NewPlasmid extends javax.servlet.http.HttpServlet implements Serializable**

**Class bioroot.plasmid.PlasmidBase extends javax.servlet.http.HttpServlet implements Serializable**

**Class bioroot.plasmid.PlasmidModify extends javax.servlet.http.HttpServlet implements Serializable**

**Class bioroot.plasmid.PlasmidSpreadSheet extends javax.servlet.http.HttpServlet implements Serializable**

**Class bioroot.plasmid.PlasmidUpload extends javax.servlet.http.HttpServlet implements Serializable**

**Class bioroot.plasmid.PlasmidViewPreferences extends javax.servlet.http.HttpServlet implements Serializable**

---

**Package** **bioroot.strain**

**Class bioroot.strain.NewStrain extends javax.servlet.http.HttpServlet implements Serializable**

**Class bioroot.strain.StrainBase extends javax.servlet.http.HttpServlet implements Serializable**

**Class bioroot.strain.StrainModify extends javax.servlet.http.HttpServlet implements Serializable**

**Class bioroot.strain.StrainSpreadSheet extends javax.servlet.http.HttpServlet implements Serializable**

**Class bioroot.strain.StrainUpload extends javax.servlet.http.HttpServlet implements Serializable**

**Class bioroot.strain.StrainViewPreferences extends javax.servlet.http.HttpServlet implements Serializable**

---

**Package** **cgar**

**Class cgar.Login extends javax.servlet.http.HttpServlet implements Serializable**

**Class cgar.TestInBioTools extends javax.servlet.http.HttpServlet implements Serializable**

---

**Package** **edu.utah.seq.data**

**Class edu.utah.seq.data.EnrichedRegion extends java.lang.Object implements Serializable**

**serialVersionUID:** 2L

**Serialized Fields**

### chromosome

```
java.lang.String chromosome
```

---

### start

```
int start
```

---

### stop

```
int stop
```

---

### numberOfWindows

```
int numberOfWindows
```

---

### bestWindow

```
SmoothingWindow bestWindow
```

---

### sequence

```
java.lang.String sequence
```

---

### treatmentPoints

```
Point[] treatmentPoints
```

---

### controlPoints

```
Point[] controlPoints
```

---

### subWindows

```
SmoothingWindow[] subWindows
```

---

### bestSubWindow

```
SmoothingWindow bestSubWindow
```

**Class edu.utah.seq.data.Info extends java.lang.Object implements Serializable**

**serialVersionUID:** 1L

**Serialized Fields**

### name

```
java.lang.String name
```

---

### versionedGenome

```
java.lang.String versionedGenome
```

:   As defined by UCSC (ie hg18, dm2, ce2, mm8), see http://genome.ucsc.edu/FAQ/FAQreleases

---

### chromosome

```
java.lang.String chromosome
```

:   As defined by UCSC see http://genome.ucsc.edu/ and particular species.

---

### strand

```
java.lang.String strand
```

:   +, -, or .

---

### readLength

```
int readLength
```

:   Length of the individual reads

---

### notes

```
java.util.HashMap<K,V> notes
```

:   Optional, can be null.

**Class edu.utah.seq.data.SmoothingWindow extends java.lang.Object implements Serializable**

**serialVersionUID:** 1L

**Serialized Fields**

### start

```
int start
```

---

### stop

```
int stop
```

---

### scores

```
float[] scores
```

**Class edu.utah.seq.data.SmoothingWindowInfo extends java.lang.Object implements Serializable**

**serialVersionUID:** 2L

**Serialized Fields**

### sm

```
SmoothingWindow[] sm
```

---

### info

```
Info info
```

---

**Package** **gata.aligner**

**Class gata.aligner.AlignerInputForm extends javax.swing.JFrame implements Serializable**

**Serialized Fields**

### panel

```
AlignerInputPanel panel
```

**Class gata.aligner.AlignerInputPanel extends javax.swing.JPanel implements Serializable**

**Serialized Fields**

### A

```
int A
```

---

### B

```
int B
```

---

### C

```
int C
```

---

### D

```
int D
```

---

### E

```
int E
```

---

### F

```
int F
```

---

### G

```
int G
```

---

### H

```
int H
```

---

### I

```
int I
```

---

### J

```
int J
```

---

### K

```
int K
```

---

### L

```
int L
```

---

### sizeTextFields

```
int sizeTextFields
```

---

### xRight

```
int xRight
```

---

### furthestRight

```
int furthestRight
```

---

### furthestDown

```
int furthestDown
```

---

### finalBaseName

```
java.lang.String finalBaseName
```

---

### stats

```
double[] stats
```

---

### refF

```
javax.swing.JTextField refF
```

---

### refBrows

```
javax.swing.JButton refBrows
```

---

### compF

```
javax.swing.JTextField compF
```

---

### compBrows

```
javax.swing.JButton compBrows
```

---

### bl2F

```
javax.swing.JTextField bl2F
```

---

### bl2FBrows

```
javax.swing.JButton bl2FBrows
```

---

### objF

```
javax.swing.JTextField objF
```

---

### objBrows

```
javax.swing.JButton objBrows
```

---

### baseNameF

```
javax.swing.JTextField baseNameF
```

---

### baseNameBrows

```
javax.swing.JButton baseNameBrows
```

---

### matSpin

```
javax.swing.JSpinner matSpin
```

---

### misSpin

```
javax.swing.JSpinner misSpin
```

---

### createSpin

```
javax.swing.JSpinner createSpin
```

---

### extSpin

```
javax.swing.JSpinner extSpin
```

---

### maskYes

```
javax.swing.JRadioButton maskYes
```

---

### maskNo

```
javax.swing.JRadioButton maskNo
```

---

### extractYes

```
javax.swing.JRadioButton extractYes
```

---

### extractNo

```
javax.swing.JRadioButton extractNo
```

---

### winTexF

```
javax.swing.JTextField winTexF
```

---

### scoreTexF

```
javax.swing.JTextField scoreTexF
```

---

### bitTexF

```
javax.swing.JTextField bitTexF
```

---

### refTexF

```
javax.swing.JTextField refTexF
```

---

### compTexF

```
javax.swing.JTextField compTexF
```

---

### help

```
javax.swing.JButton help
```

---

### reset

```
javax.swing.JButton reset
```

---

### go

```
javax.swing.JButton go
```

---

### chooser

```
javax.swing.JFileChooser chooser
```

---

### chooserDirectories

```
javax.swing.JFileChooser chooserDirectories
```

---

### frame

```
javax.swing.JFrame frame
```

---

### ap

```
AlignerPreferences ap
```

---

### gata

```
GATAligner gata
```

**Class gata.aligner.AlignerPreferences extends java.lang.Object implements Serializable**

**Serialized Fields**

### refSeq

```
java.lang.String refSeq
```

---

### compSeq

```
java.lang.String compSeq
```

---

### bl2SeqProg

```
java.lang.String bl2SeqProg
```

---

### storageLoc

```
java.lang.String storageLoc
```

---

### baseName

```
java.lang.String baseName
```

---

### match

```
java.lang.Integer match
```

---

### misMatch

```
java.lang.Integer misMatch
```

---

### create

```
java.lang.Integer create
```

---

### extend

```
java.lang.Integer extend
```

---

### mask

```
java.lang.String mask
```

---

### extract

```
java.lang.String extract
```

---

### window

```
java.lang.String window
```

---

### score

```
java.lang.String score
```

---

### refStart

```
java.lang.String refStart
```

---

### compStart

```
java.lang.String compStart
```

**Class gata.aligner.Alignment extends java.lang.Object implements Serializable**

**Serialized Fields**

### seq1

```
java.lang.String seq1
```

---

### startSeq1

```
int startSeq1
```

---

### stopSeq1

```
int stopSeq1
```

---

### seq2

```
java.lang.String seq2
```

---

### startSeq2

```
int startSeq2
```

---

### stopSeq2

```
int stopSeq2
```

---

### score

```
int score
```

---

### bitScore

```
double bitScore
```

---

### expect

```
java.lang.String expect
```

---

### orientation

```
int orientation
```

---

### AP

```
AlignParams AP
```

---

### coordinates

```
int[] coordinates
```

---

### oriWord

```
java.lang.String oriWord
```

---

### shapes

```
java.util.ArrayList<E> shapes
```

---

### LA

```
LocalAlignment LA
```

---

### realStartRef

```
double realStartRef
```

---

### realStartComp

```
double realStartComp
```

---

### relStartSeq1

```
double relStartSeq1
```

---

### relStartSeq2

```
double relStartSeq2
```

---

### relStopSeq1

```
double relStopSeq1
```

---

### relStopSeq2

```
double relStopSeq2
```

---

### relativeCoordinates

```
int[] relativeCoordinates
```

---

### LAMBDA

```
double LAMBDA
```

---

### K

```
double K
```

---

### EFF\_M

```
double EFF_M
```

---

### EFF\_N

```
double EFF_N
```

---

### color

```
java.awt.Color color
```

---

### lineColor

```
java.awt.Color lineColor
```

---

### refSeqRec

```
java.awt.geom.Rectangle2D refSeqRec
```

---

### compSeqRec

```
java.awt.geom.Rectangle2D compSeqRec
```

---

### line

```
java.awt.geom.Line2D line
```

---

### compVisible

```
boolean compVisible
```

**Class gata.aligner.AlignParams extends java.lang.Object implements Serializable**

**Serialized Fields**

### WIN\_SIZE

```
int WIN_SIZE
```

---

### MATCH

```
int MATCH
```

---

### MISMATCH

```
int MISMATCH
```

---

### GAP\_CREATE

```
int GAP_CREATE
```

---

### GAP\_EXT

```
int GAP_EXT
```

---

### MIN\_SCORE

```
int MIN_SCORE
```

---

### START\_INDEX\_REFSEQ

```
int START_INDEX_REFSEQ
```

---

### START\_INDEX\_COMPSEQ

```
int START_INDEX_COMPSEQ
```

---

### LENGTH\_REFSEQ

```
int LENGTH_REFSEQ
```

---

### LENGTH\_COMPSEQ

```
int LENGTH_COMPSEQ
```

---

### refSeqFile

```
java.lang.String refSeqFile
```

---

### compSeqFile

```
java.lang.String compSeqFile
```

---

### NAME\_REFSEQ

```
java.lang.String NAME_REFSEQ
```

---

### NAME\_COMPSEQ

```
java.lang.String NAME_COMPSEQ
```

---

### bl2seq

```
java.lang.String bl2seq
```

---

### baseName

```
java.lang.String baseName
```

---

### PATH\_RESULTS

```
java.lang.String PATH_RESULTS
```

---

### DUST

```
boolean DUST
```

---

### EXTRACT

```
boolean EXTRACT
```

---

### compVisible

```
boolean compVisible
```

---

### LAMBDA

```
double LAMBDA
```

---

### K

```
double K
```

---

### H

```
double H
```

---

### EFF\_M

```
double EFF_M
```

---

### EFF\_N

```
double EFF_N
```

**Class gata.aligner.LocalAlignment extends java.lang.Object implements Serializable**

**Serialized Fields**

### seq1

```
java.lang.String seq1
```

---

### startSeq1

```
int startSeq1
```

---

### stopSeq1

```
int stopSeq1
```

---

### seq2

```
java.lang.String seq2
```

---

### startSeq2

```
int startSeq2
```

---

### stopSeq2

```
int stopSeq2
```

---

### ori

```
int ori
```

---

### score

```
int score
```

---

### bitScore

```
double bitScore
```

---

### expect

```
java.lang.String expect
```

---

### oriWord

```
java.lang.String oriWord
```

---

### LAMBDA

```
double LAMBDA
```

---

### K

```
double K
```

---

### EFF\_M

```
double EFF_M
```

---

### EFF\_N

```
double EFF_N
```

---

**Package** **gata.geneGlyphs**

**Class gata.geneGlyphs.GlyphPanel extends javax.swing.JPanel implements Serializable**

**Serialized Fields**

### gataParams

```
GATAParams gataParams
```

---

### annoParams

```
AnnoSpecParams annoParams
```

---

### g2

```
java.awt.Graphics2D g2
```

---

### scaleBar

```
ScaleBar scaleBar
```

---

### genericGlyphs

```
GenericGlyph[][] genericGlyphs
```

---

### geneGlyphs

```
GeneRepGlyph[] geneGlyphs
```

---

### console

```
Console console
```

---

### viewPort

```
javax.swing.JViewport viewPort
```

---

### thinStroke

```
java.awt.BasicStroke thinStroke
```

---

### normalStroke

```
java.awt.BasicStroke normalStroke
```

---

### fatStroke

```
java.awt.BasicStroke fatStroke
```

---

### dashStroke

```
java.awt.BasicStroke dashStroke
```

---

### scaleBarStroke

```
java.awt.BasicStroke scaleBarStroke
```

---

### trackStrokes

```
java.awt.BasicStroke[] trackStrokes
```

---

### font

```
java.awt.Font font
```

---

### DNAColor

```
java.awt.Color DNAColor
```

---

### RNAColor

```
java.awt.Color RNAColor
```

---

### proteinColor

```
java.awt.Color proteinColor
```

---

### labelColor

```
java.awt.Color labelColor
```

---

### scaleLineColor

```
java.awt.Color scaleLineColor
```

---

### normalDNALines

```
java.awt.geom.Line2D.Double[] normalDNALines
```

---

### thinDNALines

```
java.awt.geom.Line2D.Double[] thinDNALines
```

---

### fatRNALines

```
java.awt.geom.Line2D.Double[] fatRNALines
```

---

### thinRNALines

```
java.awt.geom.Line2D.Double[] thinRNALines
```

---

### dottedProteinLines

```
java.awt.geom.Line2D.Double[] dottedProteinLines
```

---

### thinProteinLines

```
java.awt.geom.Line2D.Double[] thinProteinLines
```

---

### scaleLines

```
java.awt.geom.Line2D.Double[] scaleLines
```

---

### labels

```
java.util.ArrayList<E> labels
```

---

### trackLabels

```
java.util.ArrayList<E>[] trackLabels
```

---

### anyGenericGlyphs

```
boolean anyGenericGlyphs
```

---

### trackVis

```
boolean[] trackVis
```

---

### trackLabelVis

```
boolean[] trackLabelVis
```

---

### numberTracks

```
int numberTracks
```

---

**Package** **gata.main**

**Class gata.main.PlotterPreferences extends java.lang.Object implements Serializable**

**Serialized Fields**

### width

```
int width
```

---

### zoomedWidth

```
int zoomedWidth
```

---

### height

```
int height
```

---

### A

```
double A
```

---

### B

```
double B
```

---

### C

```
double C
```

---

### D

```
double D
```

---

### E

```
double E
```

---

### F

```
double F
```

---

### pixBtwProtRNA

```
double pixBtwProtRNA
```

---

### pixBtwTransGrps

```
double pixBtwTransGrps
```

---

### pixBtwTransGrpsGene

```
double pixBtwTransGrpsGene
```

---

### pixBtwTransGrpLabel

```
double pixBtwTransGrpLabel
```

---

### pixBtwTracks

```
double pixBtwTracks
```

---

### pixBtwLabelTrack

```
double pixBtwLabelTrack
```

---

### pixBtwDNATracks

```
double pixBtwDNATracks
```

---

### pixHeightArrows

```
double pixHeightArrows
```

---

### pixThinLineThickness

```
float pixThinLineThickness
```

---

### pixFatLineThickness

```
float pixFatLineThickness
```

---

### pixNormalLineThickness

```
float pixNormalLineThickness
```

---

### backgroundColor

```
java.awt.Color backgroundColor
```

---

### labelColor

```
java.awt.Color labelColor
```

---

### scaleLineColor

```
java.awt.Color scaleLineColor
```

---

### DNAColor

```
java.awt.Color DNAColor
```

---

### RNAColor

```
java.awt.Color RNAColor
```

---

### proteinColor

```
java.awt.Color proteinColor
```

**Class gata.main.SimpleTextWindow extends javax.swing.JFrame implements Serializable**

**Class gata.main.TextWindow extends javax.swing.JFrame implements Serializable**

**Serialized Fields**

### editorPane

```
javax.swing.JEditorPane editorPane
```

---

**Package** **gata.menu**

**Class gata.menu.FileInputForm extends javax.swing.JFrame implements Serializable**

**Serialized Fields**

### panel

```
FileInputPanel panel
```

**Class gata.menu.FileInputPanel extends javax.swing.JPanel implements Serializable**

**Serialized Fields**

### x

```
int x
```

---

### y

```
int y
```

---

### alignerFile

```
javax.swing.JTextField alignerFile
```

---

### gffFileRef

```
javax.swing.JTextField gffFileRef
```

---

### gffFileComp

```
javax.swing.JTextField gffFileComp
```

---

### frame

```
javax.swing.JFrame frame
```

---

### chooser

```
javax.swing.JFileChooser chooser
```

---

### maxWidth

```
int maxWidth
```

---

### maxHeight

```
int maxHeight
```

---

### params

```
GATAParams params
```

**Class gata.menu.GATAFrame extends javax.swing.JFrame implements Serializable**

**Serialized Fields**

### gataParams

```
GATAParams gataParams
```

---

### refAnnotation

```
Annotation refAnnotation
```

---

### compAnnotation

```
Annotation compAnnotation
```

---

### refParams

```
AnnoSpecParams refParams
```

---

### compParams

```
AnnoSpecParams compParams
```

---

### gffRefPres

```
boolean gffRefPres
```

---

### gffCompPres

```
boolean gffCompPres
```

---

### refGenericGlyphs

```
boolean refGenericGlyphs
```

---

### compGenericGlyphs

```
boolean compGenericGlyphs
```

---

### refGlyphPanel

```
GlyphPanel refGlyphPanel
```

---

### compGlyphPanel

```
GlyphPanel compGlyphPanel
```

---

### mAnnotation

```
javax.swing.JMenu mAnnotation
```

---

### conservedSeqs

```
ConservedSeqs conservedSeqs
```

---

### alignPanel

```
AlignPanel alignPanel
```

---

### plotterPreferences

```
PlotterPreferences plotterPreferences
```

---

### gataFrame

```
GATAFrame gataFrame
```

---

### genParams

```
AnnoSpecParams genParams
```

---

### documentWindow

```
TextWindow documentWindow
```

---

### innerDividerLoc

```
int innerDividerLoc
```

**Class gata.menu.SaveImageForm extends javax.swing.JFrame implements Serializable**

**Serialized Fields**

### panel

```
SaveImagePanel panel
```

**Class gata.menu.SaveImagePanel extends javax.swing.JPanel implements Serializable**

**Serialized Fields**

### x

```
int x
```

---

### y

```
int y
```

---

### saveFolderField

```
javax.swing.JTextField saveFolderField
```

---

### nameField

```
javax.swing.JTextField nameField
```

---

### widthField

```
javax.swing.JTextField widthField
```

---

### frame

```
javax.swing.JFrame frame
```

---

### chooser

```
javax.swing.JFileChooser chooser
```

---

### maxWidth

```
int maxWidth
```

---

### maxHeight

```
int maxHeight
```

---

### params

```
GATAParams params
```

---

### alignPanel

```
AlignPanel alignPanel
```

---

### saveVisible

```
boolean saveVisible
```

---

**Package** **gata.plotter**

**Class gata.plotter.AlignPanel extends javax.swing.JPanel implements Serializable**

**Serialized Fields**

### A

```
double A
```

---

### B

```
double B
```

---

### C

```
double C
```

---

### D

```
double D
```

---

### E

```
double E
```

---

### ntLenBig

```
double ntLenBig
```

---

### ntLenSmall

```
double ntLenSmall
```

---

### ntLenRefSeq

```
double ntLenRefSeq
```

---

### ntLenCompSeq

```
double ntLenCompSeq
```

---

### ntPerPixel

```
double ntPerPixel
```

---

### refRealStart

```
double refRealStart
```

---

### compRealStart

```
double compRealStart
```

---

### windowSize

```
double windowSize
```

---

### maxScore

```
double maxScore
```

---

### minScore

```
double minScore
```

---

### maxScoreColor

```
double maxScoreColor
```

---

### minScoreColor

```
double minScoreColor
```

---

### alignments

```
Alignment[] alignments
```

---

### visableAlignments

```
Alignment[] visableAlignments
```

---

### AP

```
AlignParams AP
```

---

### params

```
GATAParams params
```

---

### refX

```
double refX
```

---

### refY

```
double refY
```

---

### refW

```
double refW
```

---

### refH

```
double refH
```

---

### compX

```
double compX
```

---

### compY

```
double compY
```

---

### compW

```
double compW
```

---

### compH

```
double compH
```

---

### ref

```
java.awt.geom.Rectangle2D ref
```

---

### comp

```
java.awt.geom.Rectangle2D comp
```

---

### lineRec

```
java.awt.geom.Rectangle2D lineRec
```

---

### selectionRec

```
java.awt.geom.Rectangle2D selectionRec
```

---

### shapes

```
java.util.ArrayList<E> shapes
```

---

### orientation

```
int orientation
```

---

### score

```
double score
```

---

### lenAlignments

```
int lenAlignments
```

---

### console

```
Console console
```

---

### alignFrameWidth

```
int alignFrameWidth
```

---

### alignFrameHeight

```
int alignFrameHeight
```

---

### tools

```
ToolsFrame tools
```

---

### viewPort

```
javax.swing.JViewport viewPort
```

---

### widthBuffImage

```
int widthBuffImage
```

---

### heightBuffImage

```
int heightBuffImage
```

---

### drawCompBox

```
boolean drawCompBox
```

---

### refParams

```
AnnoSpecParams refParams
```

---

### gffRefPresent

```
boolean gffRefPresent
```

---

### compParams

```
AnnoSpecParams compParams
```

---

### gffCompPresent

```
boolean gffCompPresent
```

---

### mousePressedPoint

```
java.awt.Point mousePressedPoint
```

---

### selectRecVis

```
boolean selectRecVis
```

---

### lenVisAligns

```
int lenVisAligns
```

**Class gata.plotter.Console extends javax.swing.JFrame implements Serializable**

**Serialized Fields**

### textArea

```
javax.swing.JTextArea textArea
```

**Class gata.plotter.MultiAlignPanel extends javax.swing.JPanel implements Serializable**

**Class gata.plotter.ToolsFrame extends javax.swing.JFrame implements Serializable**

**Serialized Fields**

### AP

```
AlignParams AP
```

---

### params

```
GATAParams params
```

---

### alignPanel

```
AlignPanel alignPanel
```

---

### panel

```
javax.swing.JPanel panel
```

---

### ntRefSeq

```
javax.swing.JLabel ntRefSeq
```

---

### ntCompSeq

```
javax.swing.JLabel ntCompSeq
```

---

### minBit

```
javax.swing.JTextField minBit
```

---

### minE

```
javax.swing.JTextField minE
```

---

### maxBit

```
javax.swing.JTextField maxBit
```

---

### maxE

```
javax.swing.JTextField maxE
```

---

### lambdaNats

```
double lambdaNats
```

---

### K

```
double K
```

---

### effectiveN

```
double effectiveN
```

---

### effectiveM

```
double effectiveM
```

---

### magicNum

```
double magicNum
```

---

### decimalFormat

```
java.text.DecimalFormat decimalFormat
```

---

### numberFormat

```
java.text.NumberFormat numberFormat
```

---

**Package** **trans.anno**

**Class trans.anno.Region extends java.lang.Object implements Serializable**

**Serialized Fields**

### chromosome

```
java.lang.String chromosome
```

---

### start

```
int start
```

---

### end

```
int end
```

---

### notes

```
java.lang.String notes
```

---

### hits

```
java.util.HashSet<E> hits
```

---

### gcContent

```
boolean[] gcContent
```

---

**Package** **trans.bkgrnd**

**Class trans.bkgrnd.BackGroundOligo extends java.lang.Object implements Serializable**

**Serialized Fields**

### sequence

```
java.lang.String sequence
```

---

### coordinates

```
int[][] coordinates
```

---

### intensityArrayLists

```
java.util.ArrayList<E> intensityArrayLists
```

---

**Package** **trans.cel**

**Class trans.cel.ControlStats extends java.lang.Object implements Serializable**

**serialVersionUID:** 0L

**Serialized Fields**

### stats

```
double[] stats
```

---

### names

```
java.lang.String[] names
```

**Class trans.cel.MultiSetQuantile extends java.lang.Object implements Serializable**

**Serialized Fields**

### value

```
float value
```

---

### chromosomeNumber

```
byte chromosomeNumber
```

---

### position

```
int position
```

---

**Package** **trans.graphics**

**Class trans.graphics.IntervalDrawFrame extends javax.swing.JFrame implements Serializable**

**Serialized Fields**

### panel

```
IntervalDrawPanel panel
```

---

### scrollPane

```
javax.swing.JScrollPane scrollPane
```

---

### contentPane

```
java.awt.Container contentPane
```

**Class trans.graphics.IntervalDrawPanel extends javax.swing.JPanel implements Serializable**

**Serialized Fields**

### font

```
java.awt.Font font
```

---

### context

```
java.awt.font.FontRenderContext context
```

---

### h

```
double h
```

---

### w

```
double w
```

---

### x

```
double x
```

---

### y

```
double y
```

---

### ySpacer

```
double ySpacer
```

---

### peakFlanks

```
int peakFlanks
```

---

### intGraphs

```
Graph[] intGraphs
```

---

### matrixMismatchGraphs

```
Graph[] matrixMismatchGraphs
```

---

### boxes

```
Box[] boxes
```

---

### bindingPeaks

```
Box[] bindingPeaks
```

---

### numIntGraphs

```
int numIntGraphs
```

---

### numMatrixMismatchGraphs

```
int numMatrixMismatchGraphs
```

---

### numBoxes

```
int numBoxes
```

---

### numBindingPeaks

```
int numBindingPeaks
```

---

### title

```
java.lang.String title
```

---

### chromosome

```
java.lang.String chromosome
```

---

### runningY

```
double runningY
```

---

### runningX

```
double runningX
```

---

### textFrame

```
TextFrame textFrame
```

---

### interval

```
Interval interval
```

---

### intervalNumber

```
int intervalNumber
```

---

### selectionRec

```
java.awt.geom.Rectangle2D selectionRec
```

---

### mousePressedPoint

```
java.awt.Point mousePressedPoint
```

---

### selectRecVis

```
boolean selectRecVis
```

**Class trans.graphics.RankedSetDrawFrame extends javax.swing.JFrame implements Serializable**

**Serialized Fields**

### panel

```
RankedSetDrawPanel panel
```

---

### scrollPane

```
javax.swing.JScrollPane scrollPane
```

---

### contentPane

```
java.awt.Container contentPane
```

---

### heightAdjuster

```
int heightAdjuster
```

**Class trans.graphics.RankedSetDrawPanel extends javax.swing.JPanel implements Serializable**

**Serialized Fields**

### font

```
java.awt.Font font
```

---

### context

```
java.awt.font.FontRenderContext context
```

---

### textFrame

```
TextFrame textFrame
```

---

### maxSideWidth

```
double maxSideWidth
```

---

### alignLeftX

```
double alignLeftX
```

---

### widthMiddle

```
double widthMiddle
```

---

### alignRightX

```
double alignRightX
```

---

### spacer

```
double spacer
```

---

### glyphHeight

```
double glyphHeight
```

---

### baseScalar

```
double baseScalar
```

---

### panelWidth

```
double panelWidth
```

---

### panelHeight

```
double panelHeight
```

---

### one

```
GenomicRegion[] one
```

---

### two

```
GenomicRegion[] two
```

---

### connectorLines

```
java.awt.geom.Line2D[] connectorLines
```

---

### rectangleIntersectingColor

```
java.awt.Color rectangleIntersectingColor
```

---

### rectangleNonIntersectingColor

```
java.awt.Color rectangleNonIntersectingColor
```

**Class trans.graphics.VirtualCelFrame extends javax.swing.JFrame implements Serializable**

**Serialized Fields**

### panel

```
VirtualCelPanel panel
```

---

### scrollPane

```
javax.swing.JScrollPane scrollPane
```

---

### contentPane

```
java.awt.Container contentPane
```

---

### celMasker

```
CelMasker celMasker
```

**Class trans.graphics.VirtualCelPanel extends javax.swing.JPanel implements Serializable**

**Serialized Fields**

### frame

```
VirtualCelFrame frame
```

---

### colors

```
java.awt.Color[] colors
```

---

### colorScalar

```
double colorScalar
```

---

### intensities

```
float[][] intensities
```

---

### lengthX

```
int lengthX
```

---

### lengthY

```
int lengthY
```

---

### bufferedImage

```
java.awt.image.BufferedImage bufferedImage
```

---

### maskedEllipses

```
java.awt.geom.Ellipse2D[] maskedEllipses
```

---

### zoom

```
double zoom
```

---

### writePNG

```
boolean writePNG
```

---

### pngFile

```
java.io.File pngFile
```

---

### maxIntensityValue

```
int maxIntensityValue
```

---

### maskValue

```
float maskValue
```

---

### selectionEllipse

```
java.awt.geom.Ellipse2D selectionEllipse
```

---

### mousePressedPoint

```
java.awt.Point mousePressedPoint
```

---

### selectionVisible

```
boolean selectionVisible
```

---

**Package** **trans.main**

**Class trans.main.BindingPeak extends java.lang.Object implements Serializable**

**Serialized Fields**

### firstPeakIndex

```
int firstPeakIndex
```

---

### leftFlankingIndex

```
int leftFlankingIndex
```

---

### rightFlankingIndex

```
int rightFlankingIndex
```

---

### peakBP

```
int peakBP
```

---

### score

```
double score
```

**Class trans.main.Interval extends java.lang.Object implements Serializable**

**Serialized Fields**

### chromosome

```
java.lang.String chromosome
```

---

### start1stOligo

```
int start1stOligo
```

---

### startLastOligo

```
int startLastOligo
```

---

### sizeOfOligoMinusOne

```
int sizeOfOligoMinusOne
```

---

### numberOfWindows

```
int numberOfWindows
```

---

### bestWindow

```
Window bestWindow
```

---

### bestSubWindow

```
SubWindow bestSubWindow
```

---

### bindingPeaks

```
BindingPeak[] bindingPeaks
```

---

### sortBy

```
double sortBy
```

---

### oligos

```
Oligo[] oligos
```

---

### celFiles

```
java.io.File[] celFiles
```

---

### numberTreatmentIntensities

```
int numberTreatmentIntensities
```

---

### numberControlIntensities

```
int numberControlIntensities
```

---

### sequence

```
java.lang.String sequence
```

---

### numberMotifHits

```
int numberMotifHits
```

---

### baseScores

```
double[] baseScores
```

---

### maxCluster

```
int maxCluster
```

---

### bestWindowScored

```
boolean bestWindowScored
```

---

### fractionIntersections

```
double[] fractionIntersections
```

---

### regionNames

```
java.lang.String[] regionNames
```

**Class trans.main.Oligo extends java.lang.Object implements Serializable**

**Serialized Fields**

### index

```
int index
```

---

### start

```
int start
```

---

### matches

```
int matches
```

---

### intensities

```
java.util.ArrayList<E> intensities
```

---

### sequence

```
java.lang.String sequence
```

---

### smoothedScore

```
double smoothedScore
```

---

### skip

```
boolean skip
```

---

### inPeak

```
boolean inPeak
```

---

### rightSlope

```
double rightSlope
```

---

### leftSlope

```
double leftSlope
```

**Class trans.main.SubWindow extends java.lang.Object implements Serializable**

**Serialized Fields**

### oligos

```
Oligo[] oligos
```

---

### medianRatio

```
double medianRatio
```

---

### index

```
int index
```

**Class trans.main.Window extends java.lang.Object implements Serializable**

**Serialized Fields**

### chromosome

```
java.lang.String chromosome
```

---

### start1stOligo

```
int start1stOligo
```

---

### startLastOligo

```
int startLastOligo
```

---

### numberOligos

```
int numberOligos
```

---

### scores

```
double[] scores
```

---

### sortBy

```
double sortBy
```

---

**Package** **trans.qc**

**Class trans.qc.ChartFrame extends javax.swing.JFrame implements Serializable**

---

**Package** **trans.roc**

**Class trans.roc.RocWindow extends java.lang.Object implements Serializable**

**Serialized Fields**

### chromosome

```
java.lang.String chromosome
```

---

### start

```
int start
```

---

### end

```
int end
```

---

### middle

```
int middle
```

---

### score

```
double score
```

---

### type

```
int type
```

**Class trans.roc.Sgr extends java.lang.Object implements Serializable**

**Serialized Fields**

### chromosome

```
java.lang.String chromosome
```

---

### position

```
int position
```

---

### score

```
double score
```

---

**Package** **trans.tpmap**

**Class trans.tpmap.IntensityFeature extends java.lang.Object implements Serializable**

**serialVersionUID:** 1L

**Serialized Fields**

### start

```
int start
```

---

### intensity

```
float intensity
```

**Class trans.tpmap.MapFeature extends java.lang.Object implements Serializable**

**serialVersionUID:** 3L

**Serialized Fields**

### chromosome

```
java.lang.String chromosome
```

---

### matches

```
int matches
```

---

### start

```
int start
```

---

### pmX

```
int pmX
```

---

### pmY

```
int pmY
```

---

### mmX

```
int mmX
```

---

### mmY

```
int mmY
```

**Class trans.tpmap.TPMapFeature extends java.lang.Object implements Serializable**

**Serialized Fields**

### sequence

```
java.lang.String sequence
```

---

### chromosome

```
java.lang.String chromosome
```

---

### position

```
int position
```

---

### PMX

```
short PMX
```

---

### PMY

```
short PMY
```

---

### MMX

```
short MMX
```

---

### MMY

```
short MMY
```

---

### intensity

```
float intensity
```

---

**Package** **util.apps**

**Class util.apps.ScatterDrawFrame extends javax.swing.JFrame implements Serializable**

**Class util.apps.ScatterDrawPanel extends javax.swing.JPanel implements Serializable**

**Serialized Fields**

### lines

```
java.awt.geom.Line2D[] lines
```

---

### numLines

```
int numLines
```

---

### recs

```
java.awt.geom.Rectangle2D[] recs
```

---

### numPts

```
int numPts
```

---

### skipZeros

```
boolean skipZeros
```

---

### logValues

```
boolean logValues
```

---

**Package** **util.apps.gump**

**Class util.apps.gump.GumpApplicationPanel extends javax.swing.JPanel implements Serializable**

**Serialized Fields**

### insetPadding

```
int insetPadding
```

---

### topBottomInset

```
java.awt.Insets topBottomInset
```

---

### leftRightInsets

```
java.awt.Insets leftRightInsets
```

---

### allSidesInset

```
java.awt.Insets allSidesInset
```

---

### frame

```
GumpDrawFrame frame
```

---

### maxHeight

```
int maxHeight
```

**Class util.apps.gump.GumpDrawFrame extends javax.swing.JFrame implements Serializable**

**Serialized Fields**

### applicationPanel

```
GumpApplicationPanel applicationPanel
```

---

### applicationScrollPane

```
javax.swing.JScrollPane applicationScrollPane
```

---

### contentPane

```
java.awt.Container contentPane
```

---

### width

```
int width
```

---

### height

```
int height
```

**Class util.apps.gump.GumpResults extends javax.swing.JFrame implements Serializable**

**Serialized Fields**

### textArea

```
javax.swing.JTextArea textArea
```

**Class util.apps.gump.GumpResultsScrollPane extends javax.swing.JScrollPane implements Serializable**

**Serialized Fields**

### textArea

```
javax.swing.JTextArea textArea
```

**Class util.apps.gump.GumpToolPanel extends javax.swing.JPanel implements Serializable**

---

**Package** **util.bio.annotation**

**Class util.bio.annotation.ExonIntron extends java.lang.Object implements Serializable**

**Serialized Fields**

### start

```
int start
```

---

### end

```
int end
```

---

### name

```
java.lang.String name
```

---

### parent

```
java.lang.String parent
```

**Class util.bio.annotation.GeneGroup extends java.lang.Object implements Serializable**

**Serialized Fields**

### name

```
java.lang.String name
```

---

### chromosome

```
java.lang.String chromosome
```

---

### start

```
int start
```

---

### end

```
int end
```

---

### type

```
java.lang.String type
```

---

### transGrps

```
TransGroup[] transGrps
```

---

### geneRep

```
GeneRep geneRep
```

---

### orientation

```
int orientation
```

---

### attributes

```
java.lang.String attributes
```

**Class util.bio.annotation.GeneRep extends java.lang.Object implements Serializable**

**Serialized Fields**

### geneGrpRef

```
GeneGroup geneGrpRef
```

---

### codingSegments

```
java.util.ArrayList<E> codingSegments
```

---

### nonCodingSegments

```
java.util.ArrayList<E> nonCodingSegments
```

---

### startATGPosition

```
int startATGPosition
```

---

### fivePrimeNonCodingRegion

```
int[] fivePrimeNonCodingRegion
```

---

### threePrimeNonCodingRegion

```
int[] threePrimeNonCodingRegion
```

---

### translationFlag

```
boolean translationFlag
```

**Class util.bio.annotation.StartStop extends java.lang.Object implements Serializable**

**Serialized Fields**

### start

```
int start
```

---

### end

```
int end
```

**Class util.bio.annotation.Transcript extends java.lang.Object implements Serializable**

**Serialized Fields**

### start

```
int start
```

---

### end

```
int end
```

---

### name

```
java.lang.String name
```

---

### orientation

```
int orientation
```

**Class util.bio.annotation.TransGroup extends java.lang.Object implements Serializable**

**Serialized Fields**

### transcript

```
Transcript transcript
```

---

### translation

```
Translation translation
```

---

### exons

```
ExonIntron[] exons
```

---

### extractedStartEndExons

```
int[][] extractedStartEndExons
```

**Class util.bio.annotation.Translation extends java.lang.Object implements Serializable**

**Serialized Fields**

### start

```
int start
```

---

### end

```
int end
```

---

### transGrpRef

```
TransGroup transGrpRef
```

---

### orientation

```
int orientation
```

---

### codingSegments

```
java.util.ArrayList<E> codingSegments
```

---

### introns

```
java.util.ArrayList<E> introns
```

---

**Package** **util.bio.cluster**

**Class util.bio.cluster.Cluster extends java.lang.Object implements Serializable**

**Serialized Fields**

### correlationCoefficient

```
double correlationCoefficient
```

---

### parentCluster

```
Cluster parentCluster
```

---

### topCluster

```
Cluster topCluster
```

---

### bottomCluster

```
Cluster bottomCluster
```

---

### totalClusters

```
double totalClusters
```

---

### name

```
java.lang.String name
```

---

### floatArrayFile

```
java.io.File floatArrayFile
```

---

### nameLabel

```
javax.swing.JLabel nameLabel
```

---

### line

```
java.awt.geom.Line2D line
```

---

### parentPoint

```
java.awt.geom.Point2D parentPoint
```

---

### nameLabelCenterYCoordinate

```
double nameLabelCenterYCoordinate
```

**Class util.bio.cluster.ClusterDrawFrame extends javax.swing.JFrame implements Serializable**

**Class util.bio.cluster.ClusterDrawPanel extends javax.swing.JPanel implements Serializable**

**Serialized Fields**

### lines

```
java.awt.geom.Line2D[] lines
```

---

### numLines

```
int numLines
```

---

### labels

```
javax.swing.JLabel[] labels
```

---

### labelIndex

```
int labelIndex
```

---

### clusters

```
Cluster[] clusters
```

---

### vhc

```
HierarchicalClustering vhc
```

---

### fileNameLineSpacer

```
double fileNameLineSpacer
```

---

### fileNameSpacer

```
double fileNameSpacer
```

---

### startingXCoordinate

```
double startingXCoordinate
```

---

### xCoordinateIncrement

```
double xCoordinateIncrement
```

---

### failColor

```
java.awt.Color failColor
```

---

### labelFont

```
java.awt.Font labelFont
```

---

### minimalCCPercent

```
double minimalCCPercent
```

---

### panelWidth

```
double panelWidth
```

---

### panelHeight

```
double panelHeight
```

---

### runningX

```
double runningX
```

---

### runningY

```
double runningY
```

---

**Package** **util.gen**

**Class util.gen.TextFrame extends javax.swing.JFrame implements Serializable**

**Serialized Fields**

### textArea

```
javax.swing.JTextArea textArea
```

---


|  |  |  |
| --- | --- | --- |
| **Overview** \| Package \| Class \| Use \| **Tree** \| **Deprecated** \| **Index** \| **Help** | |  |
| PREV   NEXT | **FRAMES**    **NO FRAMES**     **All Classes** |  |


---
